# The Effects of a Single Oral Dose of Pyridoxine on Alpha-Aminoadipic Semialdehyde, Piperideine-6-Carboxylate, Pipecolic Acid, and Alpha-Aminoadipic Acid Levels in Pyridoxine-Dependent Epilepsy

**DOI:** 10.3389/fped.2019.00337

**Published:** 2019-08-26

**Authors:** Junjuan Wang, Jiao Xue, Pan Gong, Minhang Wu, Wenshuang Yang, Shiju Jiang, Ye Wu, Yuwu Jiang, Yuehua Zhang, Tatiana Yuzyuk, Hong Li, Zhixian Yang

**Affiliations:** ^1^Department of Epidemiology & Bio-Statistics, Zhejiang University School of Public Health, Zhejiang, China; ^2^Zhejiang Biosan Biochemical Technologies Co., Ltd., Zhejiang, China; ^3^Department of Pediatrics, Peking University First Hospital, Beijing, China; ^4^Department of Clinical Laboratory, Peking University First Hospital, Beijing, China; ^5^Department of Pathology, University of Utah, Salt Lake, UT, United States; ^6^ARUP Laboratories, ARUP Institute for Clinical and Experimental Pathology, Salt Lake, UT, United States; ^7^Department of Human Genetics, School of Medicine, Emory University, Atlanta, GA, United States

**Keywords:** pyridoxine-dependent epilepsy, *ALDH7A1*, pyridoxine, lysine, liquid chromatography-mass spectrometry

## Abstract

**Purpose:** To evaluate the effects of a single oral dose of pyridoxine on lysine metabolites including α-aminoadipic semialdehyde (a-AASA), piperideine-6-carboxylate (P6C), the sum of AASA and P6C (AASA-P6C), pipecolic acid (PA), and α-aminoadipic acid (α-AAA) in PDE patients.

**Methods:** The lysine metabolites of 15 patients with molecularly confirmed PDE were detected before and 4 h after taking a single oral dose of pyridoxine, respectively, using liquid chromatography-mass spectrometry (LC-MS/MS) method. Five types of samples were freshly prepared, including plasma, serum, dried blood spots (DBS), urine, and dried urine spots (DUS).

**Results:** All the patients had been treated with long-term oral pyridoxine for several months to years, with doses of 30–360 mg/d. The concentrations of a-AASA, P6C, AASA-P6C, PA, and a-AAA before and after taking a single oral dose of pyridoxine for the same analyte detected in the same type of sample varied among patients. The mean concentrations increased in almost all the metabolites after taking an oral dose of pyridoxine, with or without statistical significance. Whereas, the metabolites concentrations might increase or decrease among different patients, or in different samples of the same patient, without a regular tendency. There was no statistical correlation between the concentrations before and after taking pyridoxine in the same type of sample for most metabolites.

**Conclusions:** No obvious relationship between the metabolite levels or concentration differences and the age, pyridoxine dose (a single oral dose and long-term maintenance dose), duration of treatment, or neurodevelopmental phenotype was found at present study. The large individual differences among patients, probably affected by various genotypes, leading to quite different effects of pyridoxine on the change degree of metabolites concentrations. Our study suggested that long-term pyridoxine treatment could control seizures rather than getting toxic lysine metabolites such as a-AASA and P6C back to normal. In the future, more therapies should be focused to alleviate the metabolites accumulation and further improve the prognosis of PDE.

## Introduction

Pyridoxine-dependent epilepsy (PDE; OMIM 266100) is a rare autosomal recessive disorder, caused by mutations of aldehyde dehydrogenase 7 A1 (*ALDH7A1*) (1). It is characterized by recurrent seizures, which is resistant to conventional antiepileptic drugs but respond well to daily pharmacologic doses of pyridoxine (2). The mutations of *ALDH7A1* abolish the activity of α-aminoadipic semialdehyde (α-AASA) dehydrogenase, affecting the conversion of α-AASA to α-aminoadipic acid (α-AAA), resulting in the metabolic intermediates within the lysine catabolic pathway, including α-AASA, piperideine-6-carboxylate (P6C) and pipecolic acid (PA), elevate in urine, plasma, and cerebrospinal fluid. The pathophysiology of PDE is thought to result from chemical condensation of P6C and pyridoxal phosphate (PLP). PLP is necessary for the action of glutamic acid decarboxylase, which participate the synthesis of the inhibitory neurotransmitter GABA (1, 3). A reduction in GABA synthesis due to this secondary deficiency of PLP is partially responsible for seizure development in PDE (1). PLP is the active form of pyridoxine, and pyridoxine supplementation could cease seizures in most patients.

However, a majority of patients with PDE still had varying degrees of psychomotor development retardation under seizure control. It was generally believed that early diagnosis and treatment with pyridoxine were important to minimize severe neurodevelopmental consequences (4, 5). Considering the possible potential toxicity of the accumulating α-AASA, P6C and PA to the neurodevelopment (6), one could speculate that a dose-response relationship between pyridoxine dose and the above metabolite levels might exist and that lower metabolite levels might improve neurodevelopmental outcome. One study reported that in five PDE patients, the one receiving the highest daily dose of pyridoxine had the lowest α-AASA (7). However, heretofore, the investigations on the relationship between metabolite level and either pyridoxine dose or neurodevelopmental phenotype were rare. Here, in order to explore the possible relationship between them, the metabolites including a-AASA, P6C, the sum of AASA and P6C (AASA-P6C), PA and α-AAA were detected before and after taking a single oral dose of pyridoxine during the course.

## Materials and Methods

### Ethics Statement

This study was approved by the Biomedical Research Ethical Committee of Peking University First Hospital, and written informed consents were obtained from the legal guardians (parents) of the children. All experiments were performed in accordance with relevant guidelines and regulations.

### Samples Preparation

A total of five types of samples from 15 patients with molecularly confirmed PDE were collected, including plasma, serum, dried blood spots (DBS), urine and dried urine spots (DUS). All samples were freshly prepared. All patients were on daily pyridoxine supplements at the time of testing, without specific diet restriction. The psychomotor development was assessed according to intelligence tests (Wechsler or Gesell intelligence scales) or clinical judgment and parents' questionnaires. Blood and urine of two time points were collected: (1) immediately after getting up, before meal and taking pyridoxine; (2) 4 h after taking a single oral dose of pyridoxine in the morning. Two types of DBS were prepared with peripheral blood with or without anticoagulant, respectively. No plasma sample after taking pyridoxine was collected from Pt 1 and Pt 9, and no serum sample was collected from Pt 14.

Control samples (1–13 years old) were collected from the patients came to our hospital for genetic generalized epilepsy, tic disorders, or simple upper respiratory infection. Only samples with normal findings on routine biochemical tests, metabolic screening, or genetic tests were included. Reference ranges of the control for plasma (*n* = 28), serum (*n* = 25), DBS (*n* = 25), urine (*n* = 25), and DUS (*n* = 25) were established.

All samples were kept on dry ice during shipment and then stored at −80°C until analysis. Lysine metabolites including a-AASA, P6C, AASA-P6C, PA, α-AAA were determined.

### The Determination of the Lysine Metabolites Using LC-MS/MS

#### Reagents

The following reagents were purchased: hydrochloric acid, 3Nin n-butanol (Regis Technologies, Inc.), methanol and acetonitrile (Meker, US), formicacid, a-AAA, PA, 5-sulfosalicylic acid dihydrate Amberlyst® 15 dry resin and allysine-ethylene acetal (AEA, Sigma), d9-PA, and d3-a-AAA (CDN Isotopes).

#### AASA-P6C Synthesis

Lacking commercially available standards, the AASA-P6C reference material (a mix of a-AASA and P6C) was synthesized from AEA using Amberlyst® 15 bead according to published procedures (8). The efficiency of the conversion of AEA to AASA-P6C and residue traces of AEA were confirmed as the published procedure (7). According to these results, the final concentration of AASA-P6C in reference material was calculated based on the assumption of 100% of synthesis efficiency. Reproducibility of AASA-P6C synthesis was evaluated by the peak areas ratio of a-AASA and P6C for the same amounts of synthesized material from the different batches on three different days.

We used 1:3 ratio to approximate a-AASA/P6C concentrations based on assumption that the ionization efficiency of a-AASA and P6C were close as the published procedure (7–9).

#### Control Samples and Calibration

Six non-zero calibrators were prepared at concentrations of 2–400 umol/L for AASA-P6C, 0.5–100 umol/L for PA, and 0.5–100 umol/L for a-AAA in buffer (2.5% BSA and 0.8% NaCl) for plasma, at the same concentrations in urine for urine and in 30% acetonitrile for DBS.

Three plasma quality controls (QC) were prepared by spiking normal plasma with AASA-P6C, a-AAA, and PA standards at low (5 mol/L), medium (20 mol/L) and high (100 mol/L) concentrations. Three DBS QC were prepared by spiking normal whole blood with the same levels of AASA-P6C, a-AAA, and PA standards as whole blood. Then these samples were spotted onto filter paper card (Protein Saver™ 903® Card, Whatman Inc., Piscayaway, NJ) and allowed to dry at room temperature for about 4 h and then stored in sealed plastic bags. Three QCs were prepared in urine at low (5 mol/L AASA-P6C, a-AAA, and PA), medium (20 mol/L AASA-P6C and AAA, 50 mol/L PA), and high (200 mol/L AASA-P6C and a-AAA, 100 mol/L PA) concentrations.

#### Detection Methods

Separation was achieved on an ACQUITY BEH-C18 column (2.1 × 50 mm, 1.7 m) at a temperature of 40°C using a linear gradient of mobile phase A (0.1% of formic acid in water) and mobile phase B (0.1% of formic acid in methanol) as follows: 0 min, 10% B (0.4 mL/min); 1.5 min, 45% B (0.3 mL/min); 2.5 min, 75% B (0.3 mL/min); 3 min, 95% B (0.3 mL/min); 4–5 min, 10% B (0.3 mL/min). The mass spectrometer was operated in positive ion mode on a Waters Xevo TQD MS/MS with a 1.2 kV capillary voltage. The source and desolvation gas temperature were 150 and 550°C, respectively. The data were collected with multiple reaction monitor (MRM).

#### Plasma, Urine, and DBS Specimens

An aliquot of 20 μL of urine was mixed with 120 μL of acetonitrile (contains 15.0 μM of d3-a-AAA and d9-PA used as the internal standard). An aliquot of 50 μL of plasma was mixed with 220 μL of acetonitrile (contains 7.5 μM of d3-a-AAA and d9-PA used as the internal standard). Three DBS (ID 3 mm) was mixed with 150 μL of 50% methanol (contains 10.0 μM of d3-a-AAA and d9-PA used as the internal standard). Then the mixture was, respectively, vortexed for 2 min, allowed to stand for 3 min, and then centrifuged at 20,000 g at 4°C for 10 min. An aliquot of supernatant was transferred to a clean tube and dried by nitrogen flow in room temperature. The residue was derivatized with 100 μl of 3 N HCl in n-butanol (v/v) at 65°C and 600 rmp for 30 min dried as described above and redissolved in 100 μL water/methanol (70:30) containing 0.1% of formic acid.

#### LOD/LOQ

The limit of detection (LOD) was determined by analyzing plasma DBS, and urine lowest control samples by diluting concentrations progressively (*n* = 6) until a minimum signal-to-noise ratio (S/N) of 3 was achieved. The limit of quantification (LOQ) for all analytes was determined as LOD until a minimum signal-to-noise ratio (S/N) of 10 was achieved.

#### Precision

Three replicates of each QC level for plasma, DBS and urine were used to calculate the intra-assay precision and the inter-assay precision evaluated as CV% by analyzing the samples over 5 days. The intra-assay precision of AASA-P6C, a-AAA, and PA in plasma DBS and urine were within 10%. The inter-assay precision of AASA-P6C, a-AAA, and PA in urine were 1.91 ~ 6.18%, 2.57 ~ 8.66%, and 2.71 ~ 5.87%, respectively. The inter-assay precision of AASA-P6C, a-AAA, and PA in plasma were 4.10 ~ 5.86%, 7.04 ~ 9.79%, and 4.04 ~ 10.32%, respectively. The inter-assay precision of AASA-P6C, a-AAA, and PA in DBS were 3.38 ~ 5.8%, 2.84 ~ 4.20%, and 4.43 ~ 5.72%, respectively.

### Statistical Analysis

All statistical analyses were completed using SPSS 16.0. The Shapiro-Wilk test was used to test whether variables were normally distributed. A Student's *t*-test (2-tailed) or Mann-Whitney *U* test was used to test differences of metabolites concentrations between PDE patients and control group. A pared *t*-test (2-tailed) was used to compare the metabolites concentrations before and after taking an oral dose of pyridoxine. The associations of the metabolites concentrations between different type of samples, or between the same type of sample before and after taking pyridoxine were analyzed by calculating the correlation coefficient, and *R*^2^ > 0.7 was considered to be strong correlation. The significance level was set at 0.05 and all tests to assess *P* values were two-sided.

## Results

### Concentrations of Metabolites Before and After Taking a Single Oral Dose of Pyridoxine

**Figure 1:**
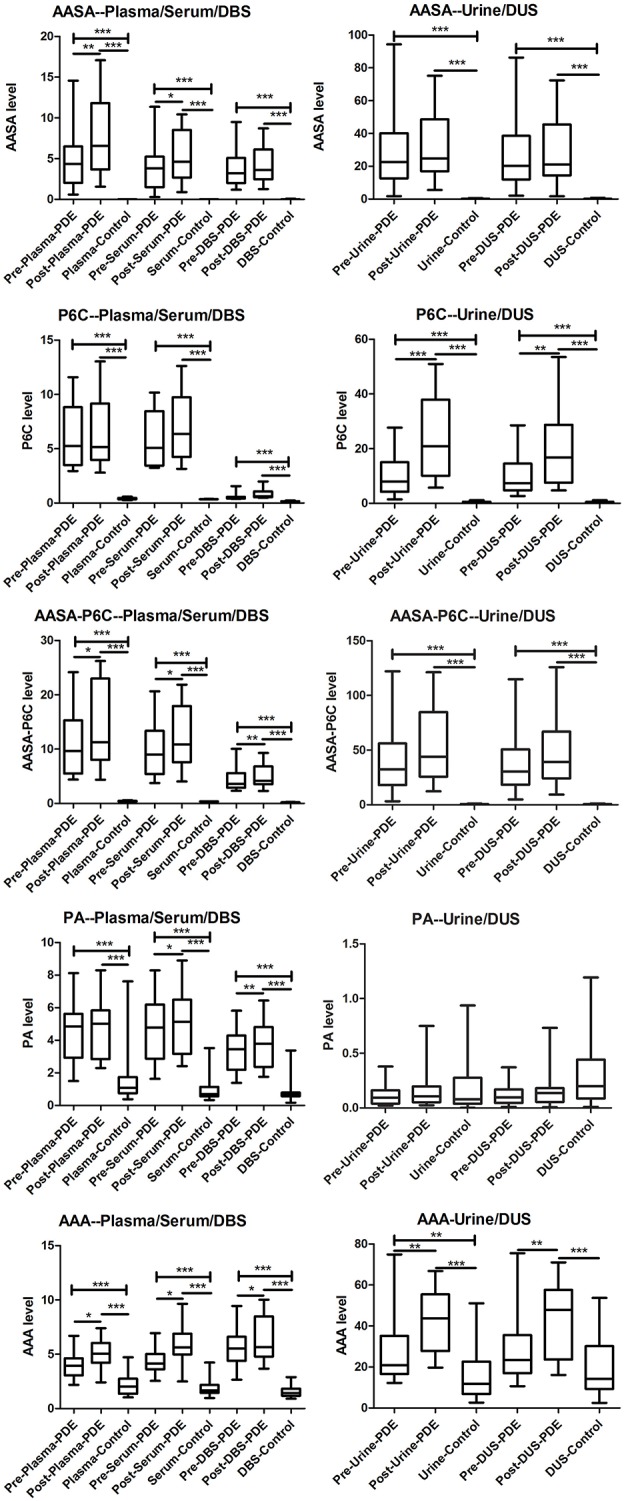
Box-plot of the a-AASA, P6C, AASA-P6C, PA, and a-AAA levels in PDE patients before and after taking a single oral dose of pyridoxine, as well as in control. ^*^P < 0.05; ^**^P < 0.01; ^***^P < 0.001.

We have measured a-AASA, P6C, AASA-P6C, PA, and a-AAA in plasma, serum, DBS, urine and DUS collected from the PDE patients before and after taking an oral dose of pyridoxine. Compared to control, in both the two time points: (1) The a-AASA, P6C and AASA-P6C in all types of samples were markedly elevated (*p* < 0.001). (2) The mean concentration of PA was elevated in plasma, serum and DBS (*p* < 0.001). (3) The mean concentration of a-AAA was elevated in all types of samples (*p* < 0.001 or *p* < 0.05), except for DUS (*p* = 0.092) before taking pyridoxine. (4) The concentration ranges of PA and a-AAA were overlapped partially between PDE and control groups, which could be moderate elevated or within normal range as specific to each PDE patient (Figure 1).

**Figure 2:**
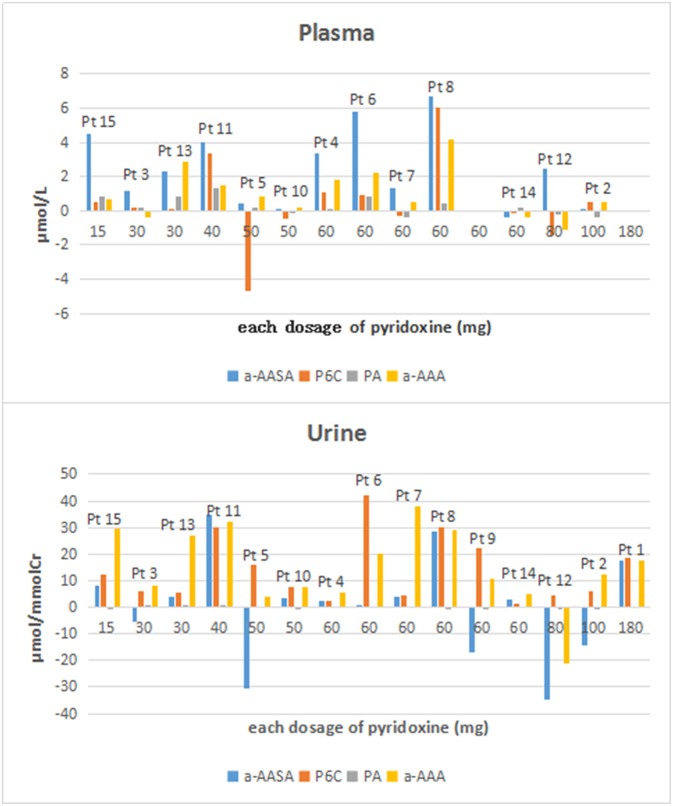
The concentration differences of a-AASA, P6C, PA, and a-AAA in plasma and urine for each patient (concentration differences = the concentrations after taking an oral dose of pyridoxine minus those before taking pyridoxine).

Compared the mean concentrations of metabolites in the samples before and after taking an oral dose of pyridoxine, we found that: (1) Significant differences of a-AASA levels were presented in plasma (*p* = 0.002) and serum (*p* = 0.023). (2) Significant differences of P6C levels were presented in urine (*p* = 0.001) and DUS (*p* = 0.005). (3) Significant differences of AASA-P6C levels were presented in plasma (*p* = 0.033), serum (*p* = 0.020), and DBS prepared with anticoagulant blood (*p* = 0.008). (4) Significant differences of PA levels were presented in serum (*p* = 0.013) and DBS prepared with anticoagulant blood (*p* = 0.007). (5) Significant differences of a-AAA levels were presented in all the five types of samples (*p* < 0.05 in all) (Table 1). The mean concentrations of the above metabolites with significant differences were higher after taking pyridoxine than before (Figure 1). However, to the single patient, the metabolites concentration after taking pyridoxine might increase or decrease among different patients, or in different samples of the same patient, without a regular tendency (Figure 2 and Figure S1).

**Table 1:** Comparison of the metabolites' mean concentrations between the samples before and after taking a single oral dose of pyridoxine.

	**a-AASA**			**P6C**			**AASA-P6C**			**PA**			**a-AAA**		
	**Pre-**	**Post-**	***P* value**	**Pre-**	**Post-**	***P* value**	**Pre-**	**Post-**	***P* value**	**Pre-**	**Post-**	***P* value**	**Pre-**	**Post-**	***P* value**
Plasma	5.1525	7.6143	**0.002**	6.0135	6.4498	0.532	11.1660	14.0641	**0.033**	4.2536	4.5357	0.076	4.1460	5.1905	**0.024**
Serum	3.8604	5.3524	**0.023**	5.9305	7.0908	0.074	9.7909	12.4432	**0.020**	4.7163	5.2218	**0.013**	4.3541	5.8941	**0.011**
DBS	3.7965	4.1943	0.053	0.6440	0.8089	0.051	4.4423	5.0032	**0.008**	3.3795	3.6717	**0.007**	5.6442	6.4438	**0.014**
Urine	31.4230	31.7325	0.951	10.5263	24.4905	**0.001**	41.9492	56.223	0.055	0.1289	0.1548	0.418	27.9851	42.9981	**0.002**
DUS	30.0522	28.1482	0.764	10.4348	19.5964	**0.005**	40.487	47.7446	0.351	0.1315	0.1598	0.438	28.5384	42.4395	**0.005**

*Bold values indicate statistical significance (p < 0.05)*.

The correlations of the concentrations in the same type of sample before and after taking an oral dose of pyridoxine were good for a-AASA (*R*^2^ = 0.898, *p* < 0.001), AASA-P6C (*R*^2^ = 0.892, *p* < 0.001), PA (*R*^2^ = 0.933, *p* < 0.001), and a-AAA (*R*^2^ = 0.718, *p* < 0.001) in DBS; good for a-AASA (*R*^2^ = 0.780, *p* < 0.001) and PA in plasma (*R*^2^ = 0.924, *p* < 0.001); and good for PA in serum (*R*^2^ = 0.898, *p* < 0.001) also (Figure S2).

### Concentrations of Metabolites Between Different Samples in PDE Patients

After taking a single oral dose of pyridoxine, the concentrations of a-AASA (*p* < 0.001) and AASA-P6C (*p* = 0.003) between the DBS prepared with anticoagulant blood and those prepared with non-anticoagulant blood were significant different, which were higher in the former. The concentrations of the a-AASA (*R*^2^ = 0.962, *p* < 0.001), AASA-P6C (*R*^2^ = 0.906, *p* < 0.001), PA (*R*^2^ = 0.894, *p* < 0.001) and a-AAA (*R*^2^ = 0.837, *p* < 0.001) in DBS from anticoagulant blood and non-anticoagulant blood after taking pyridoxine were significant positively correlated, whereas, the correlation of P6C between these two samples was not so good (*R*^2^ = 0.668, *p* < 0.001).

At both the time points before and after taking a single oral dose of pyridoxine, the concentrations of a-AASA, P6C, AASA-P6C, PA, and a-AAA between plasma and serum were positively correlated, as well as between urine and DUS. Except for P6C and a-AAA, the concentrations of a-AASA, PA and AASA-P6C in plasma and DBS of anticoagulant blood, and/or in plasma and DBS of non-anticoagulant blood were positively correlated also. The P6C level in plasma was much higher than that in DBS in both the samples before and after taking pyridoxine (*p* < 0.001).

### Evaluation the Relationships of Other Factors and Metabolites Concentrations

At the time of testing, the mean age of our 15 patients was 4.5 years old (range: 1.3–8.6 years old). The seizures in all but one patient had been controlled well with long-term oral pyridoxine for months to years (4–62 months), with doses of 30–360 mg/d (1.8–18 mg/kg/d) divided into two or three measures (15–180 mg for an oral dose). The concentrations of a-AASA, P6C, AASA-P6C, PA, and a-AAA in plasma, serum, DBS as well as in urine and DUS, varied considerably in our patients. No age-related or dose-related pattern was found between the metabolites concentrations and age, daily pyridoxine dosage and the duration of long-term oral pyridoxine (Figures S3–S5). Neurodevelopmental evaluation showed 10 out of 15 patients have expressive speech delay, most with mild delay (7/10), two with moderate delay and one with severe delay. Only 2 patients showed motor delay, all others were normal on motor development (Table 2). No correlation was found between the metabolites concentrations and psychomotor development also (Figure S6).

**Table 2:** The general information of the fifteen PDE patients.

**Patient**	**Seizure onset age**	**Age at test**	**Pyridoxine dose**	***ALDH7A1* mutations (NM_001182.4)**	**Development**	
					**Language**	**Motor**
1	3 months	4y3 months	180 mg bid (18.0 mg/kg/d)	c.1061A >G; Deletion of exon 8–13	Moderate	Normal
2	3.5 months	8y7 months	100 mg bid (7.0 mg/kg/d)	c.1553G > C; c.1061A > G	Normal	Normal
3	23 days	3y4 months	30 mg tid (5.0 mg/kg/d)	c.1279G > C; c.1279G > C	Mild	Normal
4	6 months	4y5 months	60 mg tid (8.9 mg/kg/d)	c.1547A > G; c.212C > T	Normal	Normal
5	2 days	3 years	50 mg tid (9.1 mg/kg/d)	c.1008+1G > A; c.796C > T	Mild	Normal
6	1 days	5y6 months	60 mg tid (8.1 mg/kg/d)	IVS17-1_7delCCACTAG+c.1566_1567delTA; c.871+5G > A	Moderate	Normal
7	1y1 months	5y4 months	60 mg tid (8.2 mg/kg/d)	c.1279G > C; c.986G > A	Mild	Normal
8	8 days	6y1 months	60 mg tid (7.7 mg/kg/d)	c.965 C > T; c.952 G > C	Normal	Normal
9	2 months	5y8 months	60 mg tid (7.9 mg/kg/d)	c.410G > A; c.1008+1G > A	Normal	Mild
10	1 days	4y5 months	50 mg tid (7.5 mg/kg/d)	c.1415+1G > T; c.871+5G > A	Mild	Normal
11	1 days	3y10 months	40 mg tid (6.8 mg/kg/d)	c.1531G > A; c.1008+1G > A	Severe	Normal
12	5 months	5y3 months	80 mg tid (10.9 mg/kg/d)	c.1061A > G; c.1008+1G > A	Mild	Normal
13	1 months	4y9 months	30 mg tid (4.3 mg/kg/d)	c.1547A > G; c.1061A > G	Mild	Mild
14	1.5 months	1y4 months	60 mg tid (12.9 mg/kg/d)	c.1547A > G; c.1547A > G	Normal	Normal
15	8 days	2y6 months	15 mg bid (1.8 mg/kg/d)	c.1547A > G; c.1072C > T	Mild	Normal

## Discussion

After the problem of seizure control was solved by pyridoxine in most patients, psychomotor development became an increasing concern in PDE. Presumably, developmental outcome was multi-factorial and might be influenced by not only the age of clinical onset, diagnosis and treatment, but also long-term pyridoxine compliance, associated brain dysgenesis possibly caused by the accumulated toxic metabolites, and so on (6, 10). Here, we detected the metabolites including a-AASA, P6C, AASA-P6C, PA, and α-AAA before and after taking a single oral dose of pyridoxine, in order to investigate the possible relationship between metabolites levels and pyridoxine supplement.

Compared the detection results before and after taking an oral dose of pyridoxine, the mean concentration of a-AAA was significant different in all the five types of samples; a-AASA, AASA-P6C, PA were significantly different in the several samples prepared with blood, and P6C was significant different in urine and DUS. These suggested that a-AAA might be more susceptible to oral pyridoxine than other metabolites no matter in which samples; a-AASA, AASA-P6C, and PA might be firstly influenced in blood after taking pyridoxine. These findings might provide new perspectives for further study of the interaction between pyridoxine metabolic pathway and lysine metabolic pathway.

The concentrations of a-AASA, P6C, AASA-P6C, PA, and a-AAA in both the time points varied considerably in our patients. No correlation was found between these metabolite concentrations and the age, long-term maintenance pyridoxine dose, or the duration of oral pyridoxine treatment. In addition, the concentration differences for the same analyte detected in the same type of sample before and after taking an oral dose of pyridoxine varied among patients. It seemed that the mean concentrations of almost all the metabolites increased after taking an oral dose of pyridoxine, with or without statistical significance. However, to the single individual, the metabolite concentrations might increase or decrease among different patients, or in different samples of the same patient, without a regular tendency. There was no statistical positive correlation between the concentrations before and after taking an oral dose of pyridoxine in the same type of sample for most metabolites. As well, no correlation was found between the concentration differences (before and after taking an oral dose of pyridoxine) and the single oral dose of pyridoxine. All above findings indicated that there were large individual differences, which might be decided by various genotypes, leading to quite difference of the metabolite levels in different PDE patients, even under the same dose of pyridoxine. Under the long-term pyridoxine treatment, the levels of metabolites were still elevated obviously, which might had been in a relative stable state and presented a transient change after taking each oral dose of pyridoxine. It suggested that long-term pyridoxine treatment could control seizures rather than getting toxic lysine metabolites such as a-AASA and P6C back to normal. This assumption might explain the limited efficacy of pyridoxine, as 75–80% of patients suffered developmental delay or intellectual disability despite seizure control (4, 11). However, the lack of detection before starting treatment with pyridoxine and other time points after taking an oral dose of pyridoxine resulted in these hypotheses remaining unproven. Recently, the combination of a lysine-restricted diet with pyridoxine and arginine supplements, known as triple therapy, showed improved biomarkers and psychomotor development in majority of reported PDE patients, and the direct correlation between lysine levels and PDE biomarkers had been reported (12, 13). However, it was not clear if various lysine intake contributes to the variation here because no specific diet was restricted in our patients in daily life or between the two time points of sample collected. Given the possible harmful effects of these accumulated metabolites on cerebral function (14), further application of lysine-restricted diet would be essential for our patients here. Besides, Crowther et al. had observed that the PA pathway might not be as important in lysine degradation as the saccharopine pathway, which might be another explanation for our results (15).

After taking a single oral dose of pyridoxine, the concentrations of a-AASA and AASA-P6C were significant different between DBS prepared with anticoagulant blood and those with non-anticoagulant blood, higher in the former. Moreover, except for P6C, the concentrations of all metabolites in these two types of DBS were significant positively correlated. These findings indicated that a-AASA and P6C were unstable and susceptible to other factors, and the anticoagulant in blood might influence the degradation of them. Besides, in the samples collected before and after taking the oral dose of pyridoxine, respectively, all the metabolites concentrations were positively correlated between plasma and DBS, except for P6C and a-AAA. And the P6C level in plasma was much higher than that in DBS. It indicated that P6C might be greatly affected in the process of preparing DBS from whole blood. Though many analytes used in newborn screening were known to be more stable in DBS compared to those in plasma, blood, or other aqueous solutions (16), it seemed not to apply to P6C. This might be because the dehydration process of the sample on the card affected the chemical and enzymatic hydrolysis of this analyte; or perhaps, the drying process at room temperature for about 4 h during the preparation of DBS might lead to the degradation of P6C, as the unstable properties of P6C at room temperature, even at −20°C had been reported (7, 17).

In conclusion, after detecting the lysine metabolites before and after taking a single oral dose of pyridoxine in PDE patients, no obvious relationship between metabolite levels or concentration differences (pre- and post- taking pyridoxine) and either pyridoxine dose (an oral dose and long-term maintenance dose), duration of treatment or neurodevelopmental phenotype was found at present study. The large individual differences among patients, probably affected by various genotypes, leading to quite different effects of pyridoxine on the change degree of metabolites concentrations, even under the same dose of pyridoxine. More therapies, such as triple therapy, should be focused to alleviate the metabolites accumulation and improve the prognosis of PDE in the future. Our current findings can provide insight and a baseline for further research on the pathophysiological consequences of antiquitin deficiency beyond the lysine catabolism defect.

Whereas, we acknowledged that the findings here were preliminary and had several limitations. (1) The number of PDE patients was too small to make any definite conclusion, more samples from PDE patients should be studied for validation in the next step. (2) All the patients had been treated with long-term pyridoxine supplements at the time of testing, lacking the data before starting pyridoxine treatment. (3) The detailed metabolic curve of pyridoxine had been unclear, so it was difficult to determine the peak time after taking a single oral dose of pyridoxine. Based on the metabolic curves of other medicines, we chose 4 h as a critical point for testing, which needed a further study to verify. (4) The study was unavoidably affected by some confounding factors, such as age, dietary intake, accurate time difference of sampling, personal status such as hungry or full, etc. More well-designed multi-center larger sample researches need to be done in the future.

## Data Availability

The raw data supporting the conclusions of this manuscript will be made available by the authors, without undue reservation, to any qualified researcher.

## Author Contributions

ZY, JW, and JX conceived and designed the experiments. JW, JX, PG, MW, WY, and SJ performed the experiments. ZY, JX, JW, TY, and HL analyzed the data. YW, YJ, YZ, TY, and HL contributed reagents, materials and analysis tools. JW and JX wrote the paper.

### Conflict of Interest Statement

MW was employed by company Zhejiang Biosan Biochemical Technologies Co., Ltd. The remaining authors declare that the research was conducted in the absence of any commercial or financial relationships that could be construed as a potential conflict of interest.

## Supplementary Material

Figure S1 Comparision of the a-AASA, P6C, AASA-P6C, PA, and a-AAA in plasma, serum, DBS, urine, and DUS before and after taking the single oral dose of pyridoxine.

Figure S2 Correlations of the metabolites concentrations between the same type of sample before and after taking pyridoxine.

Figure S3 Correlations between the age and metabolites concentrations.

Figure S4 Correlations between the daily dose of pyridoxine and metabolites concentrations.

Figure S5 Correlations between the duration of oral pyridoxine and metabolites concentrations.

Figure S6 Correlations between the psychomotor development and metabolites concentrations.

## References

[1] Mills PB Struys E Jakobs C Plecko B Baxter P Baumgartner M. Mutations in antiquitin in individuals with pyridoxine-dependent seizures. Nat Med. (2006) 12:307–9. 10.1038/nm1366 16491085

[2] Baxter P. Pyridoxine-dependent and pyridoxine-responsive seizures. Dev Med Child Neurol. (2001) 43:416–20. 10.1017/S0012162201000779 11409832

[3] Plecko B Paul K Paschke E Stoeckler-Ipsiroglu S Struys E Jakobs C. Biochemical and molecular characterization of 18 patients with pyridoxinedependent epilepsy and mutations of the antiquitin (ALDH7A1) gene. Hum Mutat. (2007) 28:19–26. 10.1002/humu.20433 17068770

[4] Basura GJ Hagland SP Wiltse AM Gospe SM Jr. Clinical features and the management of pyridoxine-dependent and pyridoxine-responsive seizures: review of 63 North American cases submitted to a patient registry. Eur J Pediatr. (2009) 168:697–704. 10.1007/s00431-008-0823-x 18762976

[5] Stockler S Plecko B Gospe SM Coulter-Mackie M Connolly M van Karnebeek C. Pyridoxine dependent epilepsy and antiquitin deficiency: clinical and molecular characteristics and recommendations for diagnosis, treatment and follow-up. Mol Genet Metab. (2011) 104:48–60. 10.1016/j.ymgme.2011.05.014 21704546

[6] Jansen LA Hevner RF Roden WH Hahn SH Jung S Gospe SM. Glial localization of antiquitin: implications for pyridoxine-dependent epilepsy. Ann Neurol. (2014) 75:22–32. 10.1002/ana.24027 24122892 PMC3945410

[7] Sadilkova K Gospe SM Hahn SH. Simultaneous determination of alpha-aminoadipic semialdehyde, piperideine-6-carboxylate and pipecolic acid by LC-MS/MS for pyridoxine-dependent seizures and folinic acid-responsive seizures. J Neurosci Methods. (2009) 184:136–41. 10.1016/j.jneumeth.2009.07.019 19631689

[8] Yuzyuk T Liu A Thomas A Wilson JE De Biase I Longo N. A novel method for simultaneous quantification of alpha-aminoadipic semialdehyde/piperideine-6-carboxylate and pipecolic acid in plasma and urine. J Chromatogr B Analyt Technol Biomed Life Sci. (2016) 1017–18:145–52. 10.1016/j.jchromb.2016.02.043 26970849

[9] Ferrer-López I Ruiz-Sala P Merinero B Pérez-Cerdá C Ugarte M. Determination of urinary alpha-aminoadipic semialdehyde by LC-MS/MS in patients with congenital metabolic diseases. J Chromatogr B Analyt Technol Biomed Life Sci. (2014) 944:141–3. 10.1016/j.jchromb.2013.10.032 24316525

[10] Bok LA Struys E Willemsen MA Been JV Jakobs C. Pyridoxine-dependent seizures in Dutch patients: diagnosis by elevated urinary alpha-aminoadipic semialdehyde levels. Arch Dis Child. (2007) 92:687–9. 10.1136/adc.2006.103192 17088338 PMC2083882

[11] Bok LA Halbertsma FJ Houterman S Wevers RA Vreeswijk C Jakobs C. Long-term outcome in pyridoxine-dependent epilepsy. Dev Med Child Neurol. (2012) 54:849–54. 10.1111/j.1469-8749.2012.04347.x 22804844

[12] Coughlin CR II van Karnebeek CD Al-Hertani W Shuen AY Jaggumantri S Jack RM. Triple therapy with pyridoxine, arginine supplementation and dietary lysine restriction in pyridoxine-dependent epilepsy: neurodevelopmental outcome. Mol Genet Metab. (2015) 116:35–43. 10.1016/j.ymgme.2015.05.011 26026794

[13] Yuzyuk T Thomas A Viau K Liu A De Biase I Botto LD. Effect of dietary lysine restriction and arginine supplementation in two patients with pyridoxine-dependent epilepsy. Mol Genet Metab. (2016) 118:167–72. 10.1016/j.ymgme.2016.04.015 27324284

[14] van Karnebeek CD Hartmann H Jaggumantri S Bok LA Cheng B Connolly M. Lysine restricted diet for pyridoxine-dependent epilepsy: first evidence and future trials. Mol Genet Metab. (2012) 107:335–44. 10.1016/j.ymgme.2012.09.006 23022070

[15] Crowther LM Mathis D Poms M Plecko B. New insights into human lysine degradation pathways with relevance to pyridoxine-dependent epilepsy due to antiquitin deficiency. J Inherit Metab Dis. (2019) 42:620–8 10.1002/jimd.12076 30767241

[16] Alfazil AA Anderson RA. Stability of benzodiazepines and cocaine in blood spots stored on filter paper. J Anal Toxicol. (2008) 32:511–5. 10.1093/jat/32.7.511 18713520

[17] Jung S Tran NT Gospe SM Hahn SH. Preliminary investigation of the use of newborn dried blood spots for screening pyridoxine-dependent epilepsy by LC-MS/MS. Mol Genet Metab. (2013) 110:237–40. 10.1016/j.ymgme.2013.07.017 23953072

