# Supplementary figures and images for: The Effects of a Single Oral Dose of Pyridoxine on Alpha-Aminoadipic Semialdehyde, Piperideine-6-Carboxylate, Pipecolic Acid, and Alpha-Aminoadipic Acid Levels in Pyridoxine-Dependent Epilepsy
Source: Front Pediatr. 2019 Aug 26;7:337. doi: 10.3389/fped.2019.00337 (PMC6718124; doi:10.3389/fped.2019.00337)

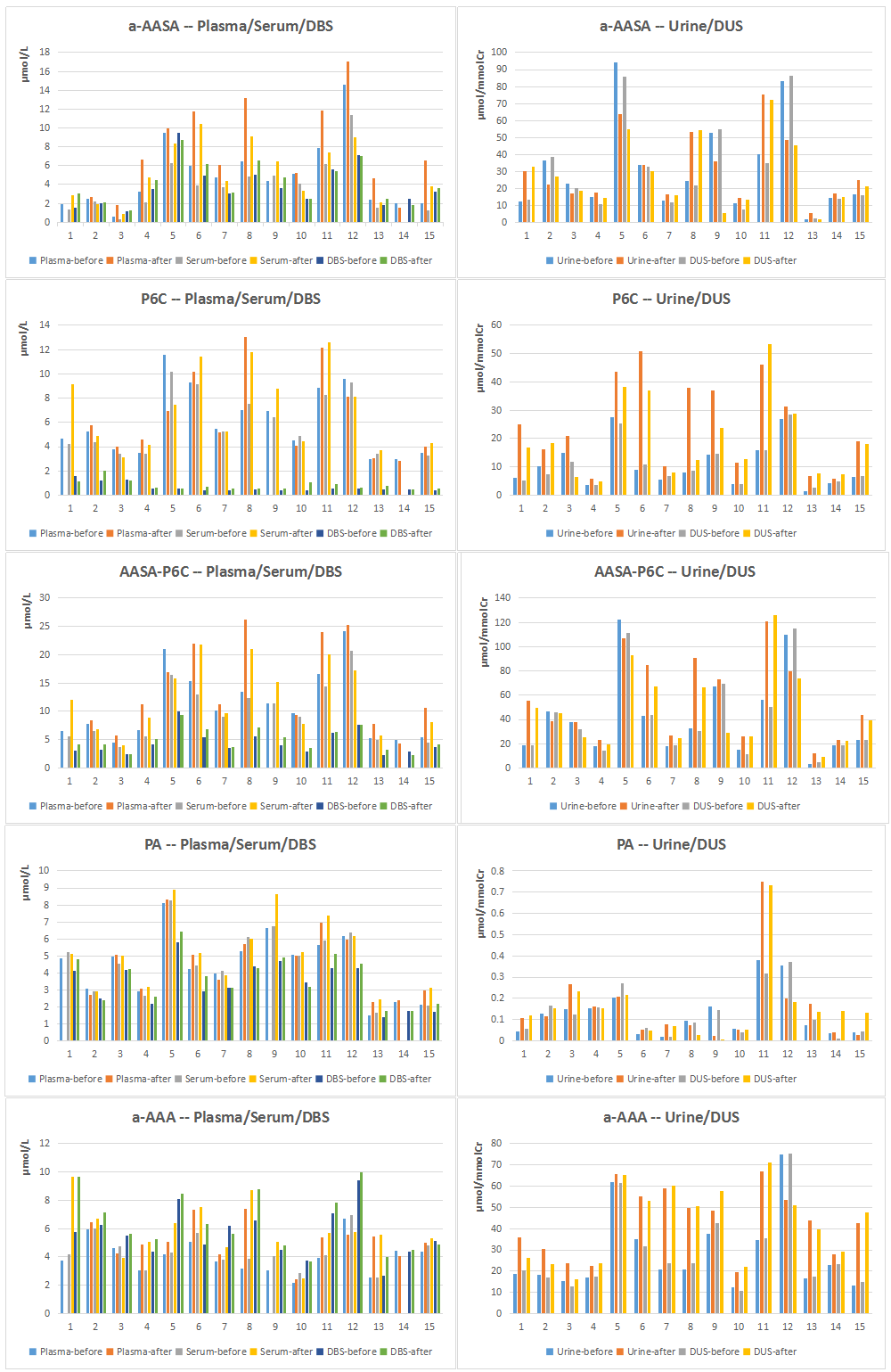

Supplement: Figure S1 — Comparision of the a-AASA, P6C, AASA-P6C, PA, and a-AAA in plasma, serum, DBS, urine, and DUS before and after taking the single oral dose of pyridoxine. [file Image_1.TIF]

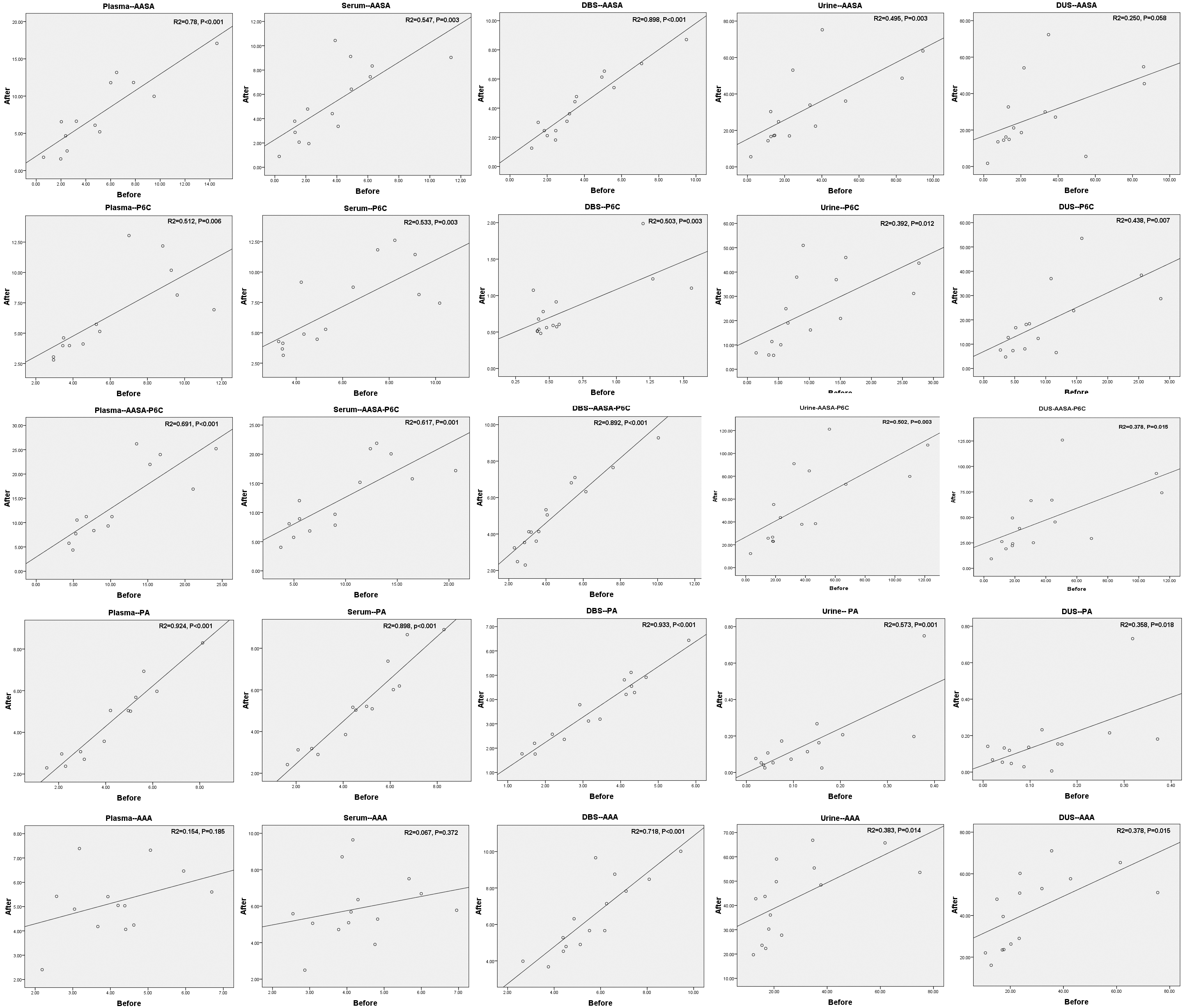

Supplement: Figure S2 — Correlations of the metabolites concentrations between the same type of sample before and after taking pyridoxine. [file Image_2.TIF]

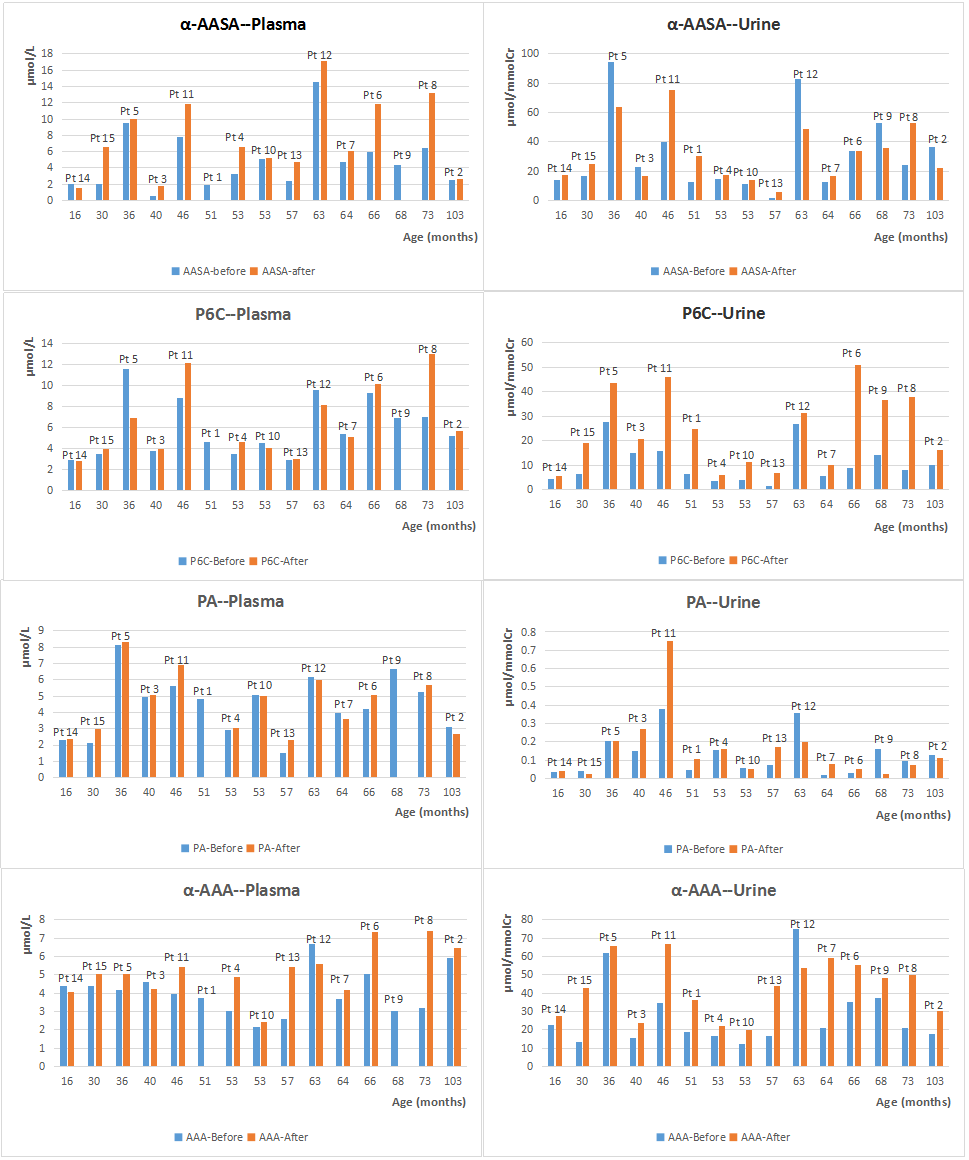

Supplement: Figure S3 — Correlations between the age and metabolites concentrations. [file Image_3.TIF]

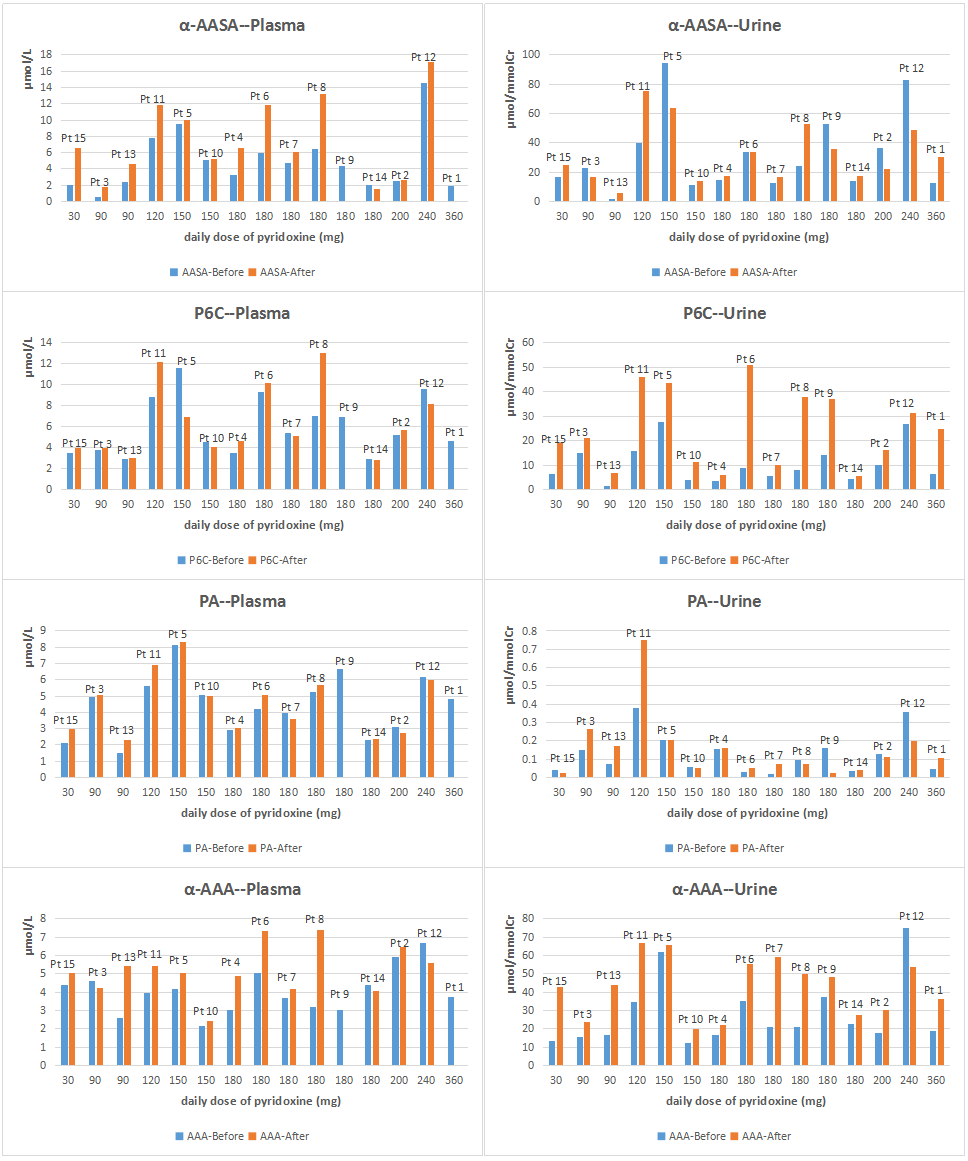

Supplement: Figure S4 — Correlations between the daily dose of pyridoxine and metabolites concentrations. [file Image_4.TIF]

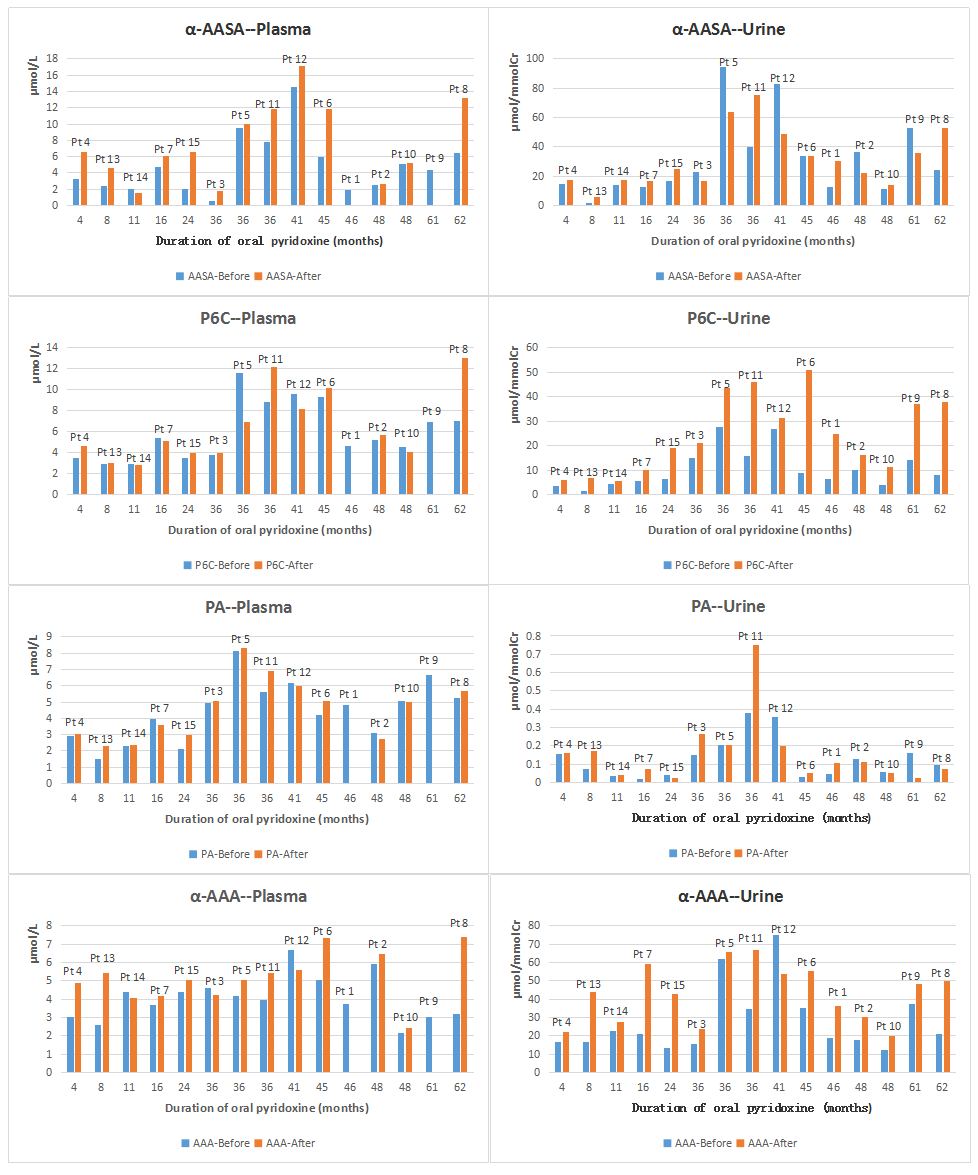

Supplement: Figure S5 — Correlations between the duration of oral pyridoxine and metabolites concentrations. [file Image_5.TIF]

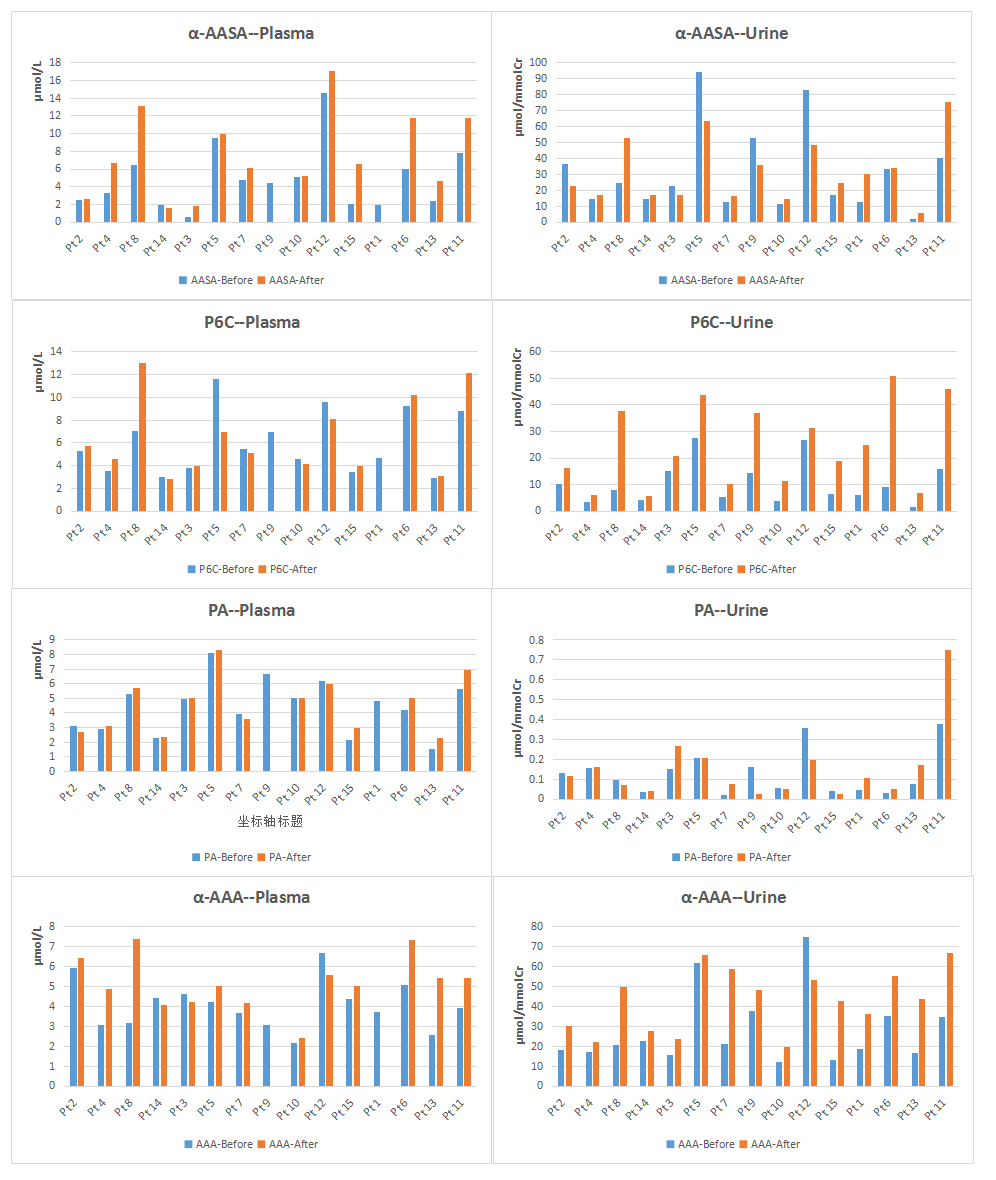

Supplement: Figure S6 — Correlations between the psychomotor development and metabolites concentrations. [file Image_6.TIF]
